# Aggressive Angiomyxoma of the Scrotum Mimicking Huge Hydrocele: Case Report and Literature Review

**DOI:** 10.1155/2009/157624

**Published:** 2009-06-25

**Authors:** Roy Morag, Eduard Fridman, Yoram Mor

**Affiliations:** ^1^Department of Urology, The Chaim Sheba Medical Center, Sackler School of Medicine, Tel-Aviv University, Tel Hashomer 52621, Israel; ^2^Department of Pathology, The Chaim Sheba Medical Center, Sackler School of Medicine, Tel-Aviv University, Tel Hashomer 52621, Israel

## Abstract

Aggressive Angiomyxoma (AAM) is a rare mesenchymal benign myxoid tumor of the pelvis and perineum which occurs almost exclusively in adult females. We are presenting a case of 64 year old male patient with a slowly growing scrotal swelling which has been regarded as hydrocele for 2 years. The patient was referred to scrotal exploration. At surgery a huge mass adjacent to the bulbar urethra was found, not involving the testicles. The morphological picture and the special stains were compatible with aggressive angiomyxoma of the scrotum and peritoneum.

## 1. Introduction

Aggressive Angiomyxoma (AAM) is a rare mesenchymal benign myxoid tumor of the pelvis and perineum which occurs almost exclusively in adult females. It is usually arising from the soft tissues of the pelvic region, perineum, vulva and buttock. Overall, its incidence is about 6-folds higher in females [1] and it is usually locally infiltrating and has a high risk of local recurrence after excision [2]. Rarely, this tumor appears in males in the scrotal, presented as a scrotal mass mimicking hydrocele or hernia [2].

## 2. Case Report

**Figure 1:**
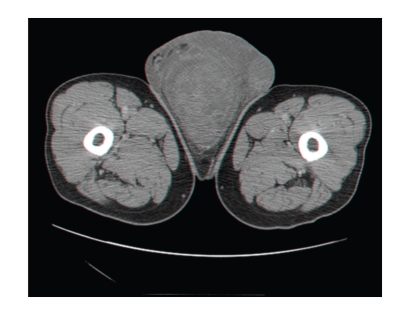
CT scan of the perineum showing a median scrotal mass.

**Figure 2:**
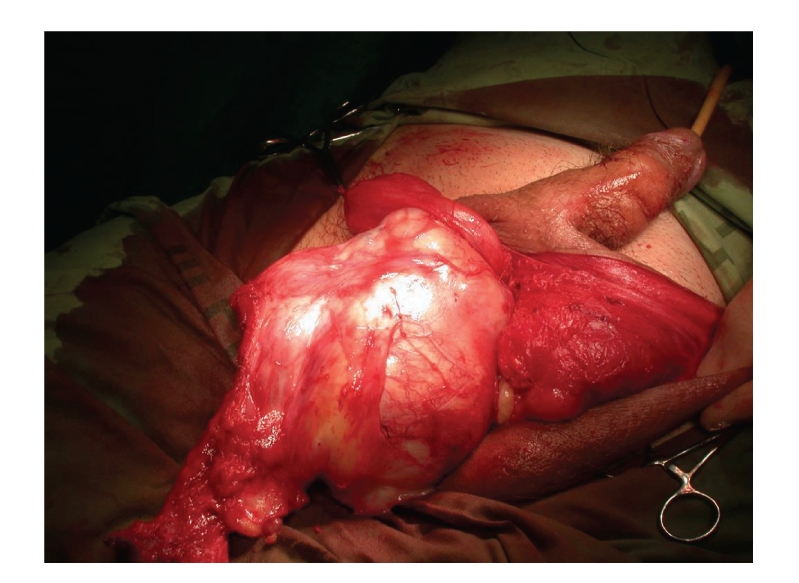
The intraoperative appearance of the scrotal mass with its relationship to the adjacent bulbar urethra.

**Figure 3:**
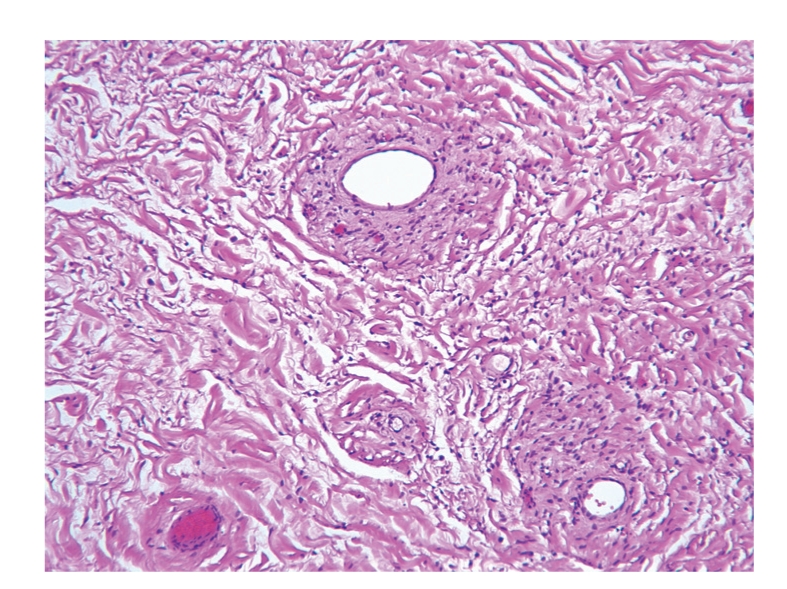
Histologic slide showing thick-walled vessels, no mitosis and collagen fibrils (H&E × 10).

**Figure 4:**
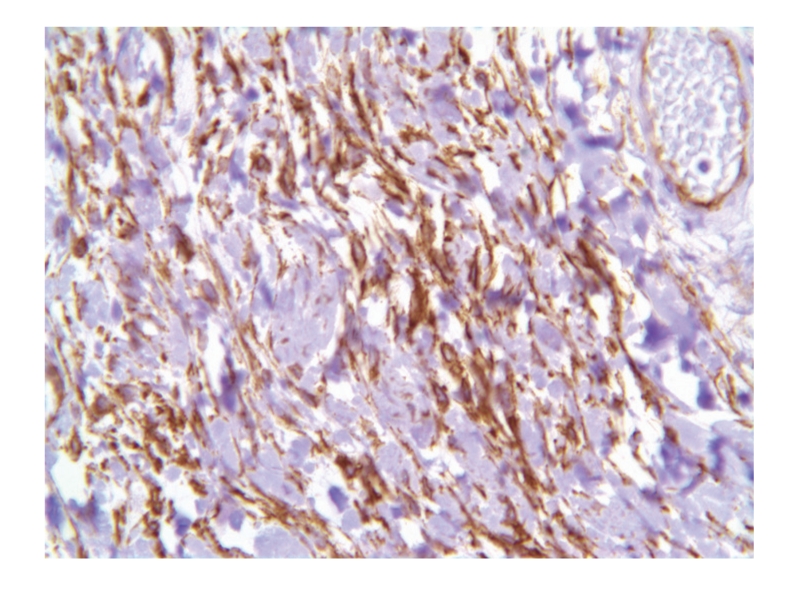
CD34 × 40 stain the spindle fiber brown.

A 64-year-old healthy patient presented with a slowly growing scrotal swelling which has been regarded as hydrocele for 2 years. Physical examination revealed a nontender scrotal swelling between the testicles which were of normal volume and consistency. Ultrasonography demonstrated diffuse median scrotal swelling accompanied with edema and small fluid pockets. Similar appearance was visualized on computed tomography (Figure 1) and the patient was subsequently referred for scrotal exploration. At surgery, a huge encapsulated lipocystic mass (7 × 11 × 19 cm/576 grams) adjacent to the bulbar urethra was found, not involving the testicles. The tumor was easily removed as it was discrete and without adhesions to the nearby tissues. Histologically, it was a paucicellular tumor composed of fibrotic and myxoid areas showing a sparse population of spindle-shaped tumor cells without cytological atypia or mitosis. Foci of thick-walled blood vessels of various sizes were identified and surrounded by edematous stromal tissue. At the periphery of the tumor, residual skeletal muscle was focally presented (Figure 2). The tumor cells were positive for CD34 (most probably myofibroblast cells Figure 3). Focal staining was present for Alpha-SMA and Desmin (supporting myoid origin of these cells), while S-100 protein was negative. Factor VIII stain highlighted numerous blood vessels. Reticulin and Masson trichome histochemical stains accentuated fibrosis and numerous blood vessels with thickened wall Figure 4. The morphological picture and the special stains were compatible with aggressive angiomyxoma of the scrotum and peritoneum. On follow-up, there were no signs of recurrence after 3 years. 

## 3. Discussion

Since 1983, when aggressive angiomyxoma was first described by Steeper and Rosai, there were about 100 cases reported worldwide (including 24 males). It often occurs in middle-aged patients ranging in age between 13 and 78 years (mean age 46). In men, AAM is usually derived from the pelviperineal interstitial tissue involving the scrotum (38%), spermatic cord (33%), perineal region (13%) and intrapelvic organs such as the bladder (8%) [1, 2]. 

AAM in the scrotal region may present as a scrotal mass, wrongly diagnosed as hernia or hydrocele, as has been previously reported in few cases [3, 4]. It is uaually difficult to identify the tumor by imaging studies such as ultrasonography and there are reports showing some advantage to scrotal MRI as it better demonstrates the angiomatous and myxomatous nature of the tumor. However, most cases are currently visualized by CT scans which are more accessible for the patients. US-guided needle biopsy has been shown to give inconclusive diagnosis [5] and the differential diagnosis includes angiomyofibroblastoma, myxoma and myxoid liposarcoma. Therefore, the final diagnosis usually awaits the final histological examination of the excisional specimen. Macroscopically, its typical appearance is of a large, grossly gelatinous and locally infiltrative tumor. The microscopic appearance of the stroma is rich of collagen, fibriles, and a prominent vascular component including many thick-walled vessels. The anomalous muscular artery is very specific for diagnosis of AAM and is the major histological difference from angiomyofibroblastoma. Immunohistochemical studies may reveal that tumor cells are immunoreactive for desmin, muscle specific actin and vimentin. Estrogen and progesteron receptor protein may be positive. The tumor cells are immunonegative for S100 protein, factor VIII related antigen, carcinoembryonic antigen and cytokeratin. Therefore, in order to differentiate the AAM from myxoma and myxoid liposarcoma the tumor cells must be positive for Vimentin and negative for S-100 protein [3, 4], as shown in the current presented case. Cytogenic analysis reveals chromosomal translocation involving chromosomes 8 and 12, associated with rearrangement of the HMGIC gene.

Surgery is the principal treatment to date and because of high risk of local recurrence (36–72%) a long-term postoperative followup with either US or CT is recommended [6, 7]. The frequent recurrence may be attributed to incomplete tumor resection and the earliest recurrence has been reported as appearing 9 months after surgery while the latest, 14 years thereafter [8]. However, no distant metastasis has been reported to date [8]. Alternatively, Poirier et al. have shown good response of the tumor to gonadotropin-relesing hormone agonist treatment which remarkably reduced the size of the tumor [6]. 

In summary, AAM is a very rare benign neoplasm which is more predominant in females. In males, scrotal AAM may be misdiagnosed as hernia or a hydrocele mainly because of the lack of awareness of this entity. 

## Figures and Tables

**Figure 5:**
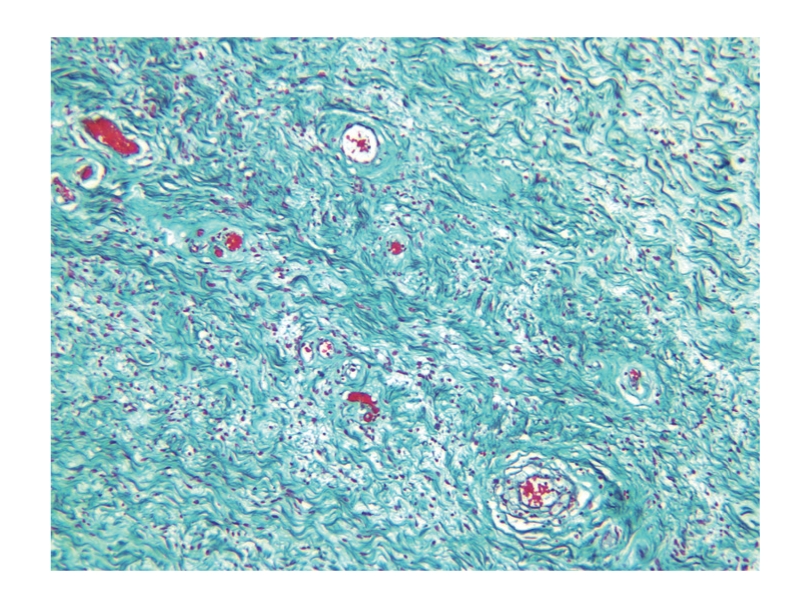
Masson × 10 very specific staining for collagen, we can also see again the thick walled blood vessels.
